# Extraforaminal lumbar interbody fusion: A systematic review of clinical outcomes, fusion rates, and safety profile

**DOI:** 10.1016/j.xnsj.2025.100830

**Published:** 2025-11-29

**Authors:** Michael K. Coffin, Kyle A. McGrath, Rebecca M. Garner, Christiana M. Cornea, Joseph S. Cheng, Justin N. Virojanapa

**Affiliations:** Department of Neurosurgery, University of Cincinnati College of Medicine, Current Institution, 231 Albert Sabin Way, PO Box, Cincinnati, OH 670515, United States

**Keywords:** Degenerative disc disease, ELIF, Extraforaminal lumbar interbody fusion, Indirect decompression, Kambin’s triangle, Lumbar fusion, Systematic review

## Abstract

**Background:**

Extraforaminal lumbar interbody fusion (ELIF) accesses the disc through a posterolateral extraforaminal corridor that preserves the posterior ligamentous complex and avoids abdominal exposure. The objective of this systematic review is to synthesize the clinical outcomes, fusion rates, complications, and surgical indications for ELIF.

**Methods:**

PRISMA 2020–conformant review registered in PROSPERO (ID: 1111090). Eligible studies reported clinical or radiographic outcomes of ELIF performed via the posterolateral extraforaminal approach. Extracted variables included indications, operative time, patient-reported outcomes, fusion, and complications. Risk of bias was assessed with the Newcastle–Ottawa Scale (NOS).

**Results:**

Thirteen retrospective studies (n=518) met inclusion. The quality of studies included was generally fair, as graded by Newcastle–Ottawa Scale (NOS). The most common indication was degenerative disc disease. Mean operative time was 168.7 minutes. Fusion by technique: open pooled mean 98%, minimally invasive pooled mean 84%, and endoscopic 100% in a single series. Pooled VAS back improved as follows: open 6.27 to 2.80 (Δ 3.47; mean follow-up 12.8 months), minimally invasive 8.16 to 3.52 (Δ 4.64; 18.8 months), endoscopic 6.49 to 1.66 (Δ 4.83; 13.2 months). Pooled VAS leg improved: open 6.66 to 2.35 (Δ 4.31, 13.3 months), minimally invasive 8.66 to 2.17 (Δ 6.49; 15.5 months), endoscopic 6.60 to 1.50 (Δ 5.10; 14.2 months). Pooled Oswestry disability index (ODI) improved: open 60.17 to 26.25 (Δ 33.92; 9.5 months), minimally invasive 56.02 to 21.46 (Δ 34.56; 18.8 months), endoscopic 34.59 to 12.19 (Δ 22.40; 13.2 months). Transient radiculopathy was reported at 9.5% and dural tear at 0.5%.

**Conclusions:**

As the first systematic review on ELIF, findings indicate it is a safe, effective alternative for lumbar fusion in select patients. Success necessitates favorable extraforaminal anatomy and intraoperative nerve monitoring to minimize complications. Future prospective trials are essential to validate these outcomes and standardize patient selection criteria.

## Background

Degenerative lumbar spine conditions, including disc herniation, foraminal stenosis, and segmental instability, represent major contributors to chronic low back pain and radiculopathy, leading to significant disability and reduced quality of life worldwide [1]. Surgical intervention, particularly spinal fusion, is often utilized for these conditions when conservative treatments fail. Posterior lumbar interbody fusion is a common approach for low-grade lumbar spondylolisthesis [2]. However, these approaches can be associated with complications and postoperative pain due to the retraction of neural structures, iatrogenic injury of posterior musculature, and disruption of the posterior ligamentous complex (PLC) [3].

Anterior and lateral alternatives, anterior lumbar interbody fusion (ALIF) and lateral lumbar interbody fusion (LLIF), reach the disc via retroperitoneal anterior and transpsoas lateral corridors, allow larger-footprint interbody devices, and can restore disc height and segmental lordosis while avoiding disruption of posterior musculature and the PLC if direct decompression with a laminectomy is not needed [4]. ALIF and LLIF carry distinct approach-specific risks: ALIF risks include vascular or visceral injury and injury to the superior hypogastric plexus, causing retrograde ejaculation in men, whereas LLIF risks arise from traversing the psoas and lumbar plexus, most commonly transient anterior-thigh sensory symptoms and hip-flexor weakness [5,6].

Extraforaminal lumbar interbody fusion (ELIF) was originally introduced by Phillips et al. [7] in 2002 under the name “intertransverse lumbar interbody fusion” as an open technique alternative to traditional anterior and posterior approaches. The technique was designed to access the disc space through a posterolateral extraforaminal corridor, more lateral than the transforaminal lumbar interbody fusion (TLIF) approach, thereby preserving the PLC, avoiding the abdominal cavity, and minimizing neural injury risk [7]. A key anatomical feature that facilitates the ELIF procedure is Kambin’s triangle, a triangular zone located laterally in the lumbar spine, bounded by the exiting nerve root, the superior articulating process, and the superior endplate of the lower vertebral body [8]. Traditionally, Kambin’s triangle has been discussed in the context of the TLIF procedure, in which case accessing this corridor necessitates disruption of the PLC and bony anatomy (laminectomy, ipsilateral facetectomy), however in the context of an ELIF, the disc space can be accessed via this corridor without disruption of the aforementioned elements, making it a relatively safe pathway for access to the disc space.

This systematic review aims to critically evaluate the current body of evidence on ELIF, with a focus on its clinical indications, technical variations, patient-reported outcomes, fusion rates, complications, and biomechanical rationale. The primary objective is to determine what is currently known about ELIF as a lumbar fusion technique and to summarize its safety, efficacy, and surgical applications across the available literature.

## Methods

### Search strategy

This review was conducted in accordance with PRISMA 2020 guidelines and was prospectively registered with PROSPERO (Registration ID:1111090). A systematic search was performed in PubMed, Embase, and Google Scholar related to ELIF in accordance with preferred reporting items for systematic reviews and meta-analysis (PRISMA) guidelines using the search term “ELIF.” One search term was used based off the most common language in the literature to describe the technique. The search was completed on July 20, 2025, with no restrictions on publication date. Reference lists of included studies were manually screened for additional eligible articles; no additional articles were found from this.

Two reviewers screened all titles, abstracts, and full texts against the inclusion criteria. All selections were independently reviewed and verified by a third individual. Discrepancies were resolved by consensus or an independent third-party member of the team when needed. The selection process is documented in a PRISMA flow diagram.

### Eligibility criteria

**Fig. 1:**
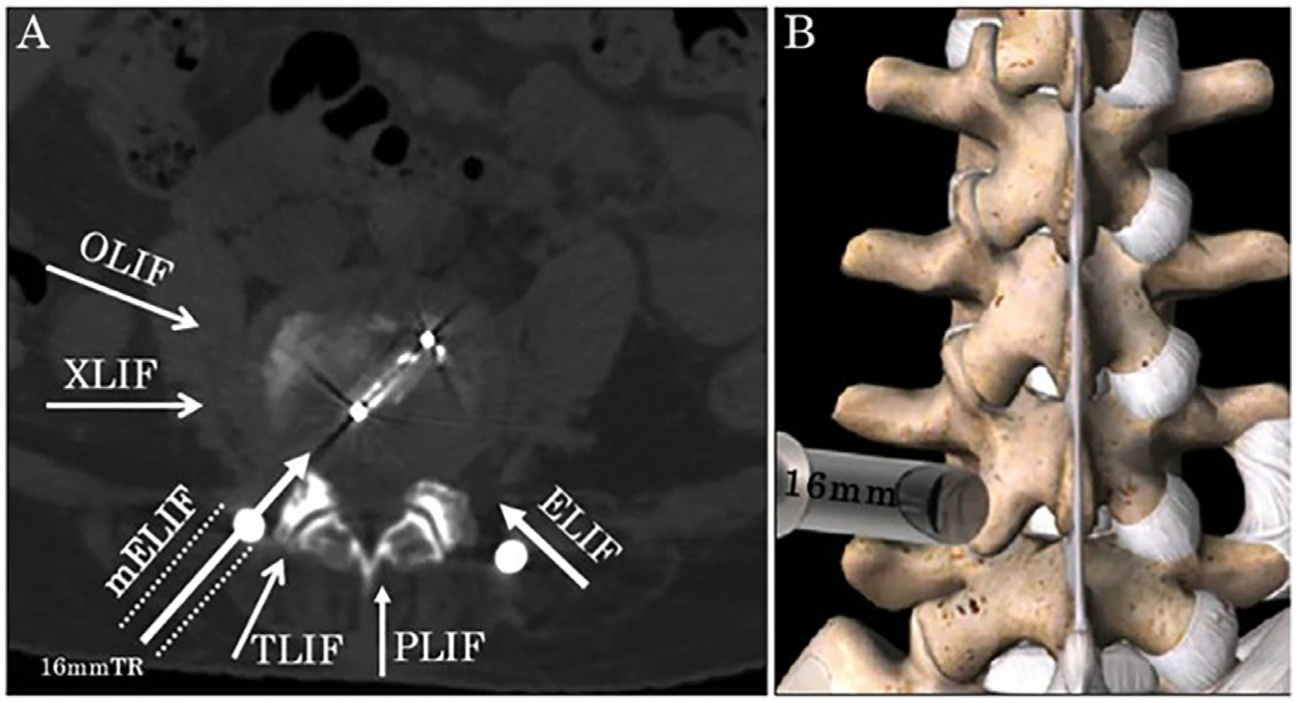
Representative postoperative images after mELIF (AP and lateral radiographs; sagittal and axial MRI; CT demonstrating fusion around the cage) (Fig. 4) [17].

**Fig. 2:**
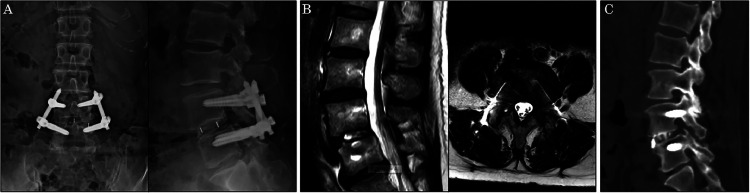
Schematic of the microendoscopy-assisted extraforaminal lumbar interbody fusion (mELIF) approach, illustrating the extraforaminal working corridor and cage trajectory (Fig. 1) [17].

Studies were eligible if they described ELIF procedures involving a posterolateral, extraforaminal approach consistent with the original intertransverse technique described by Phillips and Cunningham[7], accessing the disc space via Kambin’s triangle or the intertransverse interval. Representative postoperative radiographs and cross-sectional image schematic of mELIF are shown for anatomical and technique context (Fig. 1, Fig. 2). Inclusion criteria included reported data on one or more of the following: visual analog scores (VAS) for pain before and after surgery, fusion rates, Oswestry disability index (ODI) scores, complications, or surgical techniques. Exclusion criteria included cadaveric or biomechanical studies, non-English articles, conference abstracts, individual case reports, reviews, and studies that did not isolate ELIF outcomes from other fusion techniques.

### Data extraction and quality assessment

All study-level data were extracted into a Microsoft Excel worksheet by one independent reviewer and independently checked by another. Extracted information included patient demographics, surgical details, outcome measures, complication rates, and follow-up intervals. For studies reporting multiple follow-up intervals, the longest was used.

Quality of studies and biases were assessed for each study using the Newcastle–Ottawa scale (NOS) for non-randomized control cohorts. Studies were assigned scores based on selection, comparability, and outcome domains, and results were summarized in narrative form. Total NOS scores were used to classify studies as good (7–9), fair (5–6), or poor quality (≤4)

### Statistical analysis

Surgical techniques, outcome definitions, and follow-up intervals varied, so a formal meta-analysis was not performed. A descriptive synthesis was conducted. Continuous outcomes were summarized as sample-size weighted pooled means and pooled standard deviations using the following equations:

$$\begin{array}{ccc} & & \text{Pooled}\;\text{mean}:\left\{\bar{x}\right\}=\frac{\left\{\sum n_{i}{\bar{x}}_{i}\right\}}{\left\{\sum n_{i}\right\}}\\ & & \text{Within}-\text{group}\;\text{sum}\;\text{of}\;\text{studies}:SS_{\left\{within\right\}}=\sum \left[\left(n_{i}-1\right)s_{i}^{2}\right]\\ & & \text{Between}-\text{group}\;\text{sum}\;\text{of}\;\text{studies}:SS_{\left\{between\right\}}=\sum n_{i}{\left({\bar{x}}_{i}-\left\{\bar{x}\right\}\right)}^{2}\\ & & \text{Pooled}\;\text{variance}:s^{\left\{2\right\}}=\frac{\left\{SS_{within}+SS_{\left\{between\right\}}\right\}}{\left\{\left(\sum n_{i}\right)-1\right\}}\\ & & \text{Pooled}\;\text{standard}\;\text{deviation}:s=\sqrt{s^{2}} \end{array}$$

Studies without standard deviations contributed to pooled means but not to pooled standard deviations. Follow-up time was summarized as a sample-size–weighted mean in months.

## Results

### Search process and quality

**Fig. 3:**
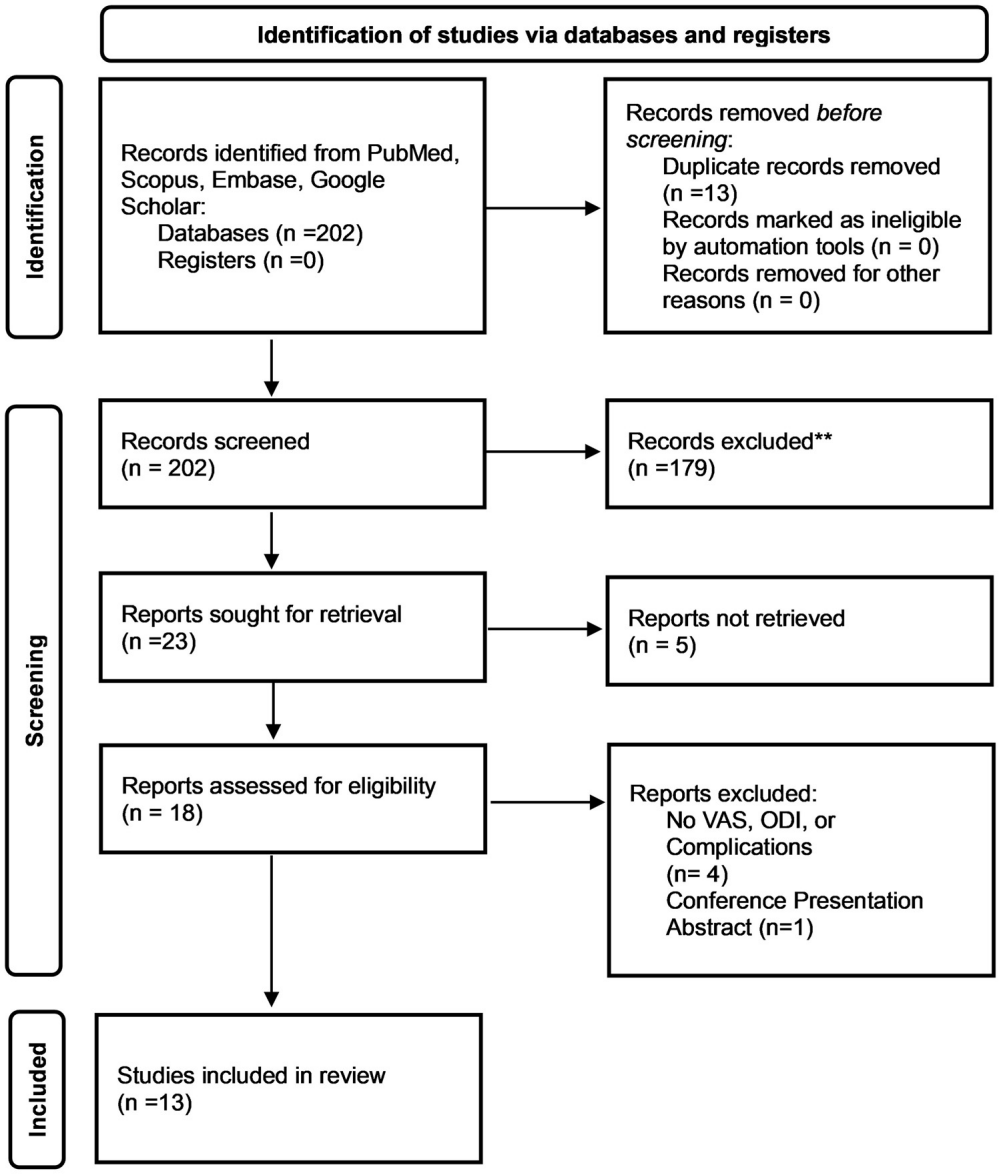
PRISMA 2020 flow diagram illustrating the study selection process for the systematic review. The initial database search identified 202 records. After removal of duplicates and screening of titles and abstracts, 13 full-text studies met inclusion criteria and were included in the final analysis. Boxes represent stages of screening, arrows indicate study flow, and numbers within boxes correspond to the number of records at each stage. PRISMA = Preferred Reporting Items for Systematic Reviews and Meta-Analyses.

The initial search identified 202 citations. After applying inclusion and exclusion criteria, 13 studies were included in this systematic review, with 179 studies excluded. All the studies included were retrospective cohort studies (Fig. 3 and Table 1).

**Table 1:** Included studies characteristics.

Author	Study type	Sample size	Age (Mean±SD)	M/F	Reason*	Levels	Technique
Lee et al. [8]	RC	12	60.7±14.6	5/7	Revision surgery	L3–L5	MIS
Lu et al. [11]	RC	16	45.3	11/5	Revision surgery	L4–5	Open
Vcelak et al. [12]	RC	82	60.45±10.57	41/41	DDD	L1–S1	Open
You et al. [13]	RC	12	63.3±8.4	8/4	DDD	L4–S1	Endoscopic
Kurzbuch et al. [14]	RC	100	47	53/47	Isthmic spondylolisthesis	L5–S1	Open
Baek and Lee, [15]	RC	5	51.8	3/2	DDD	L4–S1	Open
Chin et al. [16]	RC	10	47.5	1/9	DDD	L4–S1	MIS
Gonzalez et al. [10]	RC	29	53.9	13/16	DDD	L5–S1	Open
Shibayama et al. 2021[10]	RC	55	62.7	17/38	DDD	L3–S1	Endoscopic
Landriel et al. [18]	RC	40	57	15/25	DDD	L2–L5	MIS
Doria et al. [19]	RC	23	53.3	13/10	DDD	L3–S1	MIS
Yoshimizu et al. [20]	RC	27	66.1±10.7	10/17	DDD	L4–5	Endoscopic
Recoules-Arche et al. [9]	RC	107	52.8	40/67	DDD	L2–S1	Open

Abbreviations: RC, retrospective study; SD, standard deviation; M, male; F, female; DDD, degenerative disc disease; MIS, minimally invasive surgery.

⁎ Most common diagnosis treated with surgery in the articles by amount in the cohort.

The methodological quality of the 13 included cohort studies was assessed using the NOS. Overall, most studies were rated as fair quality, with final scores ranging from 4 to 9. One study used the criteria for good quality, scoring highly across all domains [9]. One study received the lowest score due to a notably short follow-up period of one month and insufficiently comparable outcomes [10]. The remaining studies generally demonstrated adequate cohort selection and outcome ascertainment, although many did not include a non-exposed comparison group or control for important confounding variables. Studies are summarized in Table 2.

**Table 2:** Quality of studies/risk of bias.

Author	Study type	NOS score	Quality tier
Lee et al. [8]	RC	6	Fair
Lu et al. [11]	RC	6	Fair
Vcelak et al. [12]	RC	6	Fair
You et al. [13]	RC	6	Fair
Kurzbuch et al. [14]	RC	6	Fair
Baek and Lee [15]	RC	6	Fair
Chin et al. [16]	RC	6	Fair
Gonzalez et al. [10]	RC	4	Poor
Shibayama et al. [10]	RC	6	Fair
Landriel et al. [18]	RC	6	Fair
Doria et al. [19]	RC	6	Fair
Yoshimizu et al. [20]	RC	6	Fair
Recoules-Arche et al. [9]	RC	8	Good

### Operative time

Operative time was reported in 10 studies [[11], [12], [13], [14], [15], [16], [17], [18], [19], [20]]. The operative durations ranged from 96.9 minutes to 338 minutes across studies. The Open cohort mean operative duration was 179.3 minutes [12,14,15], 153.7 minutes for the MIS cohort [16,18,20], and 152.8 minutes for the Endoscopic cohort [13,17,20].

### Fusion rates

Fusion rates were reported in 8 studies [8,9,11,14,16,[18], [19], [20]]. The Open ELIF cohort had a pooled mean fusion rate of 98% (range: 97%–100%) [9,11,14]. The MIS cohort had a pooled mean fusion rate of 84% (range: 60%–100%) [8,16,18,19]. The single study utilizing an endoscopic approach reported a fusion rate of 100% [20]. Studies are summarized in Table 3.

**Table 3:** Fusion rate in studies.

Author	Fusion rates	Technique
Recoules-Arche et al. [9]	97%	Open
Lu et al. [11]	100%	Open
Kurzbuch et al. [14]	99%	Open
Pooled mean	98%	
		
Chin et al. [16]	60%	MIS
Landriel et al. [18]	77.5%	MIS
Doria et al. [19]	95.7%	MIS
Lee et al. [8]	100%	MIS
Pooled mean	84%	
		
Yoshimizu et al. [20]	100%	Endoscopic
		

### Visual analog scale and Oswestry disability index

Thirteen studies reported preoperative and postoperative visual analog scale (VAS) scores for back pain [[8], [9], [10], [11], [12], [13], [14], [15], [16], [17], [18], [19], [20]]. The Endoscopic cohort decreased from a mean of 6.49 to 1.66 (Δ 4.83) over a mean follow-up of 13.2 months [13,17,20]. The MIS cohort decreased from a mean of 8.16 to 3.52 (Δ 4.64) over a mean follow-up of 18.8 months [8,16,18,19]. The Open cohort decreased from a mean of 6.27 to 2.80 (Δ 3.47) [[9], [10], [11], [12],^14^,^15^,]. Studies are summarized in Table 4.

**Table 4:** Visual analog scale back values (Mean±SD).

Author	Pre-operation	Post-operation	Follow-up (months)	Technique
Lu et al. [11]	6.3±2.9	2.6±0.8	12	Open
Vcelak et al. [12]	5.60±0.83	3.16±0.81	12	Open
Recoules-Arche et al. [9]	4.48±3.0	2.34±2.8	24	Open
Kurzbuch et al. [14]	8	3	5	Open
Baek and Lee [15]	8	5.4	12.2	Open
Gonzalez et al. [10]	8.51	2.41	1	Open
Pooled mean	6.27±2.45	2.80±2.13	12.79	
				
Lee et al. [8]	6.43±1.04	3.04±0.71	27.1±3.2	MIS
Chin et al. [16]	7.8	4.8	24	MIS
Landriel et al. [18]	8.81±0.62	2.12±0.89	12	MIS
Doria et al. [19]	8.09	5.65	24	MIS
Pooled mean	8.16±1.25	3.52±0.93	18.79	
				
Yoshimizu et al. [20]	7.7±2.1	1.9±2.2	31	Endoscopic
Shibayama et al. [17]	5.9±2.45	1.6±1.66	6	Endoscopic
You et al. [13]	6.5±1.8	1.4±0.9	6	Endoscopic
Pooled mean	6.49±2.39	1.66±1.75	13.18	
				

VAS scores for leg pain were reported in 8 studies [8,9,12,14,15,17,18,20]. The MIS group decreased from a preoperative mean of 8.66 to 2.17 (Δ 6.49) [8,18]. The Open group decreased from a mean of 6.66 to 2.35 (Δ 4.31) [9,12,14,15], and the Endoscopic group decreased from a mean of 6.60 to 1.50 (Δ 5.10) [17,20]. Studies are summarized in Table 5.

**Table 5:** Visual analog scale leg values (Mean±SD).

Author	Pre-operation	Post-operation	Follow-up (months)	Technique
Vcelak et al. [12]	6.04±1.45	2.44±0.71	12	Open
Kurzbuch et al. [14]	7	2	5	Open
Baek and Lee, [15]	7	1.2	12.2	Open
Recoules-Arche et al. [9]	6.81±2.9	2.65±2.9	24	Open
Pooled mean	6.66±2.41	2.35±2.23	13.3	
				
Lee et al. [8]	7.70±0.70	2.91±0.73	27.1±3.2	MIS
Landriel et al. [18]	8.95±0.36	1.95±0.65	12	MIS
Pooled mean	8.66±0.70	2.17±0.78	15.48	
				
Yoshimizu et al. [20]	7.4±2.3	1.5±2.3	31	Endoscopic
Shibayama et al. [17]	6.2±2.42	1.5±1.69	6	Endoscopic
Pooled mean	6.60±2.43	1.50±1.90	14.23	

Eleven studies reported ODI scores [[8], [9], [10], [11], [12], [13],[16], [17], [18], [19], [20]]. Preoperative mean ODI scores were 60.17 for the Open group [[10], [11], [12]], 56.02 for the MIS group [8,16,18,19], and 34.59 for the Endoscopic group [13,17,20]. Postoperative mean scores were 26.25 for the Open group (Δ 33.92) [[10], [11], [12]], 21.46 for the MIS group (Δ 34.56) [8,16,18,19], and 12.19 for the Endoscopic group (Δ 22.40) [13,17,20]. Studies are summarized in Table 6.

**Table 6:** Oswestry disability index values (Mean±SD).

Author	Pre-operation	Post-operation	Follow-up (months)	Technique
González et al. [10]	45.27±10.65	15.65±10.28	1	Open
Lu et al. [11]	70.9±15.3	14.6±4.9	12	Open
Vcelak et al. [12]	63.35±8.62	32.27±11.61	12	Open
Pooled mean	60.17±13.16	26.25±13.40	9.49	
				
Chin et al. [16]	53	24.4	24	MIS
Lee et al. [8]	76.78±6.08	29.91±2.98	27.1±3.2	MIS
Landriel et al. [18]	51.9±4.96	12.2±3.19	12	MIS
Doria et al. [19]	53.65	31.87	24	MIS
Pooled mean	56.02±11.78	21.46±8.15	18.79	
				
Yoshimizu et al. [20]	53.6±18.7	14.9±16.2	31	Endoscopic
You et al. [13]	54.2±11.6	21.4±9.4	6	Endoscopic
Shibayama et al. [17]	20.98±5.04	8.85±5.26	6	Endoscopic
Pooled mean	34.59±19.81	12.19±10.93	13.18	

### Complications

Ten studies reported postoperative complications [8,[11], [12], [13], [14],[16], [17], [18], [19], [20]]. Transient radiculopathy was the most commonly observed complication. Dural tears were reported in 3 studies [11,13,18], and cage migration in 2 studies [17,20]. Superficial wound infections occurred in 2 studies [8,20]. One study reported a revision surgery rate of 11.1%, with 3.7% of patients requiring early reoperation for worsening neurological symptoms [12]. Another reported transient neuropathic pain in 15% of patients [20]. Three studies reported no complications. [9,10,15].

## Discussion

### Fusion outcomes

Fusion rates across the included studies varied widely, ranging from 60% to 100% [8,9,11,14,16,[18], [19], [20]], placing ELIF within the ranges reported for other lumbar interbody fusion techniques: ALIF 97.8%, PLIF 91.4%, TLIF 96%, and LLIF 85.6% [21,22]. Several factors likely contributed to this variability, including differences in surgical technique, patient selection, and the classification system used for fusion assessment. For instance, Recoules-Arche et al. [9] reported a 97% fusion rate in a large cohort of 107 patients using an ELIF technique with unilateral pedicle screws and rods, attributing their high rate to the large surface area of their two cages. Doria et al. [19] similarly documented near-complete fusion at 97.5% at 6 months with structured follow-up protocols, which they attributed to their endplate preparation and intertransverse grafting.

In contrast, Chin et al. [16] reported only a 60% fusion rate, possibly attributable to limited visualization and technical constraints of their percutaneous oblique approach. These findings underscore that while ELIF can achieve fusion rates comparable to other interbody techniques, its outcomes are highly contingent upon technical execution.

### VAS and ODI improvements

Most studies reported significant postoperative improvements in both axial and radicular pain [8,9,[11], [12], [13], [14], [15], [16],^10^,[17], [18], [19], [20]], as well as functional disability [8,9,[10], [11], [12], [13],^16^,[17], [18], [19], [20]]. For comparison, reported mean ODI reductions for other lumbar interbody fusion techniques are approximately 32 points for ALIF, 31.1 for PLIF, 39.2 for TLIF, and 32.2 for LLIF, with corresponding mean change VAS for back scores at follow-up averaging 5.0 for ALIF, 5.5 for PLIF, 4.3 for TLIF, and 5.6 for LLIF [21,23,24]. These outcomes support ELIF as a viable alternative to established lumbar interbody fusion techniques, with comparable improvements in pain and disability observed at an average follow-up of approximately 15 months.

The clinical improvements observed following ELIF are likely attributable not only to interbody stabilization but also to indirect neural decompression. By restoring disc height, ELIF reduces posterior disc bulging and places tension on the hypertrophied ligamentum flavum, effectively pulling it taut and thinning its profile. These changes collectively contribute to decompression of the thecal sac and exiting nerve roots [10,[12], [13], [14], [15], [16], [17], [18], [19], [20]]. This process results in enlargement of the spinal canal and foramina without direct posterior decompression. The procedure employs a posterolateral access with an anteromedial working trajectory, targeting the disc space through the extraforaminal corridor. Although one comparative study involving biportal endoscopic ELIF utilized an expandable cage to enhance distraction [20], the remaining studies relied on static implants.

### Complications

Complication rates across ELIF studies were generally low, although variability existed depending on surgical technique and patient characteristics [8,[11], [12], [13], [14],[16], [17], [18], [19], [20]]. In our pooled analysis, the most frequent complication was transient radiculopathy, occurring in approximately 9.5% of patients. This may be explained by the anatomical position of the dorsal root ganglion (DRG) within the extraforaminal working corridor and its heightened mechanosensitivity to compression [25,26]. Retraction of the exiting nerve root during ELIF can exert pressure on the DRG, producing localized inflammation and resulting in sensory or motor deficits. The predominance of transient symptoms in the included studies supports an inflammatory rather than structural injury mechanism.

Intraoperative monitoring of the exiting nerve root is commonly used to mitigate this complication, and in other monitored cohorts within our review, the incidence of transient radiculopathy was generally lower than the overall average (9.5%) [10,18]. Notably, Chin et al. [16] reported a neurological complication rate of 50% despite the use of intraoperative neurophysiology monitoring, substantially higher than any other included study. The next highest rate was 16%, reported by Recoules-Arche et al. [9], with most other series well below this threshold. The elevated rate in Chin et al. may be attributable to their use of a percutaneous minimally invasive approach, which was not employed by any other study in our review. Most importantly, the absence of any stated prior clinical experience with ELIF in their report could also explain this

Chin et al. [16] also acknowledged the morphometric findings of Bae et al. [27], who demonstrated that in the lower lumbar spine, the extraforaminal “danger zone” containing the lumbar trunk and exiting nerve roots can extend up to 25 mm anteriorly from the intervertebral foramen as a possible explanation for their high complication rate. They also stated that the working corridor becomes wider at more cranial lumbar levels [16]. However, cadaveric evidence from Hoshide et al [28] and Kumari et al. [29] reported a progressive increase in Kambin’s triangle area from L1–L2 to L4–L5. These discrepancies underscore the variability in anatomical descriptions and measurement methodologies across studies, which may contribute to differences in reported complication rates

When considered in the context of other lumbar interbody fusion techniques, ELIF’s complication profile appears to be within range at 13.45%. TLIF has reported overall complication rates ranging from 13% to 17%, while PLIF ranges from 12% to 16.1% [21,30]. Dural leakage occurs in approximately 3.7% of posterior approaches compared to 0.5% in ELIF [21]. Neurological deficits were reported in 7.3% of PLIF and 4% of TLIF procedures [21]. LLIF, in contrast, is associated with lumbar plexus–related complications, with transient sensory or motor deficits reported in 13% to 38% of cases [[31], [32], [33], [34]]. Taken together, the available evidence indicates that ELIF’s complication rates align with those of established interbody fusion approaches while avoiding certain high-impact complications seen in anterior, lateral, and posterior techniques, in particular dural leaks.

### Biomechanical and technique advantages

Lumbar interbody fusion accesses the disc space through Kambin’s triangle, allowing dorsolateral cage placement on the apophyseal ring while preserving midline posterior elements and the ventral annulus [35]. This engages structurally stronger bone, shifting load toward lateral and ventral zones and away from the weaker central endplate [36], a configuration expected to reduce subsidence risk and provide a stable fusion environment. Cadaveric and finite element studies show that this placement increases lateral and ventral strain under extension and limits lateral bending and axial rotation more effectively than TLIF with identical unilateral fixation [37,38], thereby minimizing micromotion at the bone–implant interface. Stability comparable to bilateral pedicle screw constructs can be achieved with supplemental fixation such as a contralateral translaminar facet screw [37,38].

ELIF should be favored when preservation of the PLC is critical, such as in younger patients or those at risk for adjacent segment degeneration. Similar to an anterior or lateral approach, ELIF allows for direct access to the disc space and therefore introduction of a larger cage footprint than a TLIF/PLIF, improving fusion surface, allowing for indirect foraminal decompression, and reducing subsidence risk [38]. Additionally, unlike posterior approaches, ELIF allows for preservation of the PLC, which plays a key role in maintaining spinal stability. Disruption of these elements has been linked to increased biomechanical stress at adjacent levels and higher rates of symptomatic adjacent segment disease over time [39]. Additionally, ELIF avoids direct manipulation of the thecal sac and minimizes muscle dissection, making it particularly suitable for revision cases with posterior scar tissue or in patients where posterior re-entry carries increased risk [40].

Compared to anterior techniques, ELIF offers a posterior-sparing alternative without the need for retroperitoneal or transpsoas access. This makes it advantageous in patients with prior abdominal surgery, complex vascular anatomy, or higher surgical risk profiles. While anterior approaches have demonstrated radiographic outcomes in terms of lordosis restoration showing improvement in sagittal alignment [3,41], ELIF cohorts reporting alignment parameters are scarce and show small, inconsistent changes but, pain reduction and function are similar. Biomechanical studies suggest that larger lordotic cages are feasible, but their clinical use for sagittal correction has not been tested. Consequently, the existing literature does not establish this as an effective primary technique for sagittal correction because the available cohorts predominantly use ELIF for indirect decompression and segmental stabilization.

### Technical considerations

Despite these benefits, ELIF presents several limitations and technical challenges. The procedure is highly dependent on the surgeon’s familiarity with the three-dimensional anatomy of the extraforaminal corridor. It requires a steep learning curve and proficiency in minimally invasive techniques, which may affect complication rates during early adoption. Furthermore, ELIF is not well-suited for addressing central canal or lateral recess stenosis, where direct decompression of the traversing nerve root is necessary [21]. Finally, large extruded or sequestered disc herniations may represent a relative contraindication for an isolated extraforaminal approach, given the limited working corridor for fragment exploration and the potential for increased nerve root manipulation. In such cases, posterior or combined approaches may be more appropriate.

Anatomical variability also poses a limitation. Intervertebral disc height decreases with age as DDD becomes more prevalent, and markedly collapsed discs can substantially narrow the working corridor for an extraforaminal approach [42]. In patients with very low preoperative disc height, the reduced disc space may function as a relative technical contraindication that must be considered during patient selection. Variability in the contents of Kambin’s triangle further compounds this constraint, because up to 20% of patients have vascular structures traversing the space, including radicular arteries or anomalous veins, which may increase the risk of bleeding and limit safe access to the disc space [43]. Preoperative imaging and careful intraoperative dissection are critical to mitigate these risks.

Accessing the L5–S1 disc space via the extraforaminal route presents unique anatomical challenges compared to more cranial lumbar levels. The overhang of the L5 transverse process, prominence of the sacral ala, and height of the iliac crest can restrict visualization and limit the working corridor, particularly in patients with a steep lumbosacral angle. To overcome these constraints, partial resection of the sacral ala or superior articular process of S1 has been described to expand access while preserving overall stability [14]. In cases with severely collapsed disc height, additional bone removal, occasionally including partial drilling of the iliac wing, has been reported to facilitate optimal cage placement and achieve adequate decompression [10]. These modifications demonstrate that L5–S1 is technically feasible for ELIF when careful preoperative imaging is used to plan for enlargement of the bony corridor.

### Limitations and future directions

This review has several limitations, consistent with common issues in spine surgery literature. All included studies were retrospective, introducing risks of selection and reporting bias. No randomized controlled trials or prospective comparative studies were available. Although all studies used CT for fusion assessment, a methodological strength, there was variability in the classification systems applied. However, all systems ultimately evaluated the same endpoint: the presence of trabecular bone bridging between vertebral bodies, minimizing the impact of this heterogeneity.

Functional outcomes were assessed using validated instruments such as VAS and ODI, but not all studies reported both measures, and some lacked complete data. The surgical technique was carried out in various ways, from open to endoscopic, throughout some of the studies included, even though the overall surgical approach was identical and not separated by type in the pooled average analysis due to the limited number of studies available. The review was restricted to English-language studies indexed in major databases, introducing potential language and publication bias. However, the risk of bias and methodological quality was systematically assessed for all included studies.

Future research should prioritize prospective study designs with standardized outcome reporting. Comparative trials, ideally randomized or propensity-matched, are needed to assess ELIF’s relative efficacy and safety against established interbody techniques such as TLIF, PLIF, ALIF, and LLIF. Uniform consensus radiographic fusion criteria will improve data synthesis and comparability. Longitudinal follow-up beyond two years is necessary to evaluate the durability of fusion, hardware integrity, and reoperation rates.

Additionally, the potential role of ELIF in sagittal alignment correction warrants further investigation. Studies incorporating lordotic cages through the posterolateral corridor should assess changes in segmental and global lumbar lordosis, exploring whether ELIF could be adapted for use in deformity correction strategies. This could be pursued through both biomechanical cadaveric studies and then radiographic analysis in clinical cohorts. Clarifying ELIF’s capacity for angular correction would expand its applicability beyond degenerative pathology and inform surgical planning in more complex spinal reconstruction.

## Conclusions

To our knowledge, this is the first systematic review to synthesize clinical outcomes, fusion rates, and complications associated with the ELIF technique. ELIF is a safe and effective alternative for lumbar fusion in select patients dealing with degenerative disc disease and low-grade spondylolisthesis who need indirect decompression. Clinical outcomes, fusion rates, and complication data support its use in cases where posterior preservation, revision access are advantageous. Optimal use hinges on selecting patients whose anatomy permits a reliable extraforaminal corridor while reserving alternative or combined strategies for less favorable morphologies. Intraoperative monitoring of the exiting nerve root may reduce transient radiculopathy and future work should standardize monitoring protocols and report complications stratified by monitoring status. Further prospective, comparative trials are needed to validate these findings and define optimal patient selection criteria.

## Funding

No funding was received.

## Declaration of competing interest

The authors declare that they have no known competing financial interests or personal relationships that could have appeared to influence the work reported in this paper.
